# Supplementary material for: A Novel Urinary miRNA Biomarker for Early Detection of Colorectal Cancer
Source: Cancers (Basel). 2022 Jan 17;14(2):461. doi: 10.3390/cancers14020461 (PMC8773893; doi:10.3390/cancers14020461)
Supplement: Supplementary file 1 [file cancers-14-00461-s001.zip › cancers-1492902-supplementary.pdf]

Supplementary Tables S1–S4

Supplementary Table S1. TaqMan Advanced miRNA Assays for qRT-PCR.

Assay Name	Assay ID	Context Sequence
hsa-miR-129-1-3p	480873_mir	AAGCCCUUACCCCAAAAAGUAU
hsa-miR-566	479373_mir	GGGCGCCUGUGAUCCCAAC
hsa-miR-598-5p	479080_mir	GCGGUGAUCCCGAUGGUGUGAGC
hsa-miR-4669	478925_mir	UGUGUCCGGGAAGUGGAGGAGG
hsa-miR-6756-5p	480284_mir	AGGGUGGGGCUGGAGGUGGGGCU

Supplementary Table S2. Urinary miRNA biomarker in the patients with well to poorly differentiated CRC.

		miR-129-1-3p ($2^{-\Delta Ct}$, Median, IQR)	*p* Value	miR-566 ($2^{-\Delta Ct}$, Median, IQR)	*p* Value
HC (*n* = 203)		0.00040 (0.00016–0.00096)		0.048 (0.026–0.140)	
CRC	tub (*n* = 186)	0.00145 (0.00086–0.00292)	< 0.001*	0.200 (0.082–0.473)	< 0.001*
	por (*n* = 17)	0.00152 (0.00081–0.00262)	< 0.001*	0.136 (0.042–0.343)	0.064*

Ct, threshold cycle; IQR, interquartile range; HC, healthy control; CRC, colorectal cancer; tub, well to moderate differentiated adenocarcinoma; por, poorly differentiated adenocarcinoma

*** *P* values were calculated in comparison with HC.**

Supplementary Table S3. Expression of miR-129-1-3p and miR-566 in serum sample.

		HC (n = 24)	CRC (n = 24)	p Value
miR-129-1-3p	$2^{-\Delta Ct}$, Median, IQR	0.00259 (0.00081–0.00756)	0.00445 (0.00292 –0.00740)	0.071
miR-566	$2^{-\Delta Ct}$, Median, IQR	0.259 (0.113–1.976)	1.304 (0.471–1.984)	0.027

HC, healthy control; CRC, colorectal cancer; IQR, interquartile range; Ct, threshold cycle;

Supplementary Table S4. Characteristics of the patients with CRC for tissue miRNA analysis.

ID	Age (years)	Gender	Location	Stage	Histological grade
CRC1	55	Male	Sigmoid	IIIB	moderately differentiated
CRC2	74	Female	Cecum	IIA	poorly differentiated
CRC3	73	Female	Sigmoid	IIA	well differentiated
CRC4	44	Male	Rectum	IIA	well differentiated
CRC5	38	Female	Sigmoid	IIA	well differentiated
CRC6	80	Female	Sigmoid	IIIB	well differentiated
CRC7	69	Female	Ascending	IIIC	poorly differentiated
CRC8	83	Female	Transverse	IIA	moderately differentiated
CRC9	68	Male	Sigmoid	IIIB	well differentiated
CRC10	69	Female	Ascending	IIA	moderately differentiated
CRC11	52	Male	Transverse	IIA	moderately differentiated
CRC12	70	Female	Sigmoid	IIIB	moderately differentiated
CRC13	73	Female	Cecum	IIIB	mucinous
CRC14	77	Male	Sigmoid	IIB	well differentiated
CRC15	78	Female	Cecum	IIA	well differentiated
CRC16	74	Male	Rectum	IIA	moderately differentiated
CRC17	72	Male	Rectum	IIA	moderately differentiated
CRC18	68	Male	Sigmoid	IIA	well differentiated
CRC19	52	Male	Ascending	IIIB	poorly differentiated
CRC20	70	Male	Ascending	IIA	well differentiated
CRC21	75	Female	Rectum	IIIB	moderately differentiated

**CRC22**	**69**	**Male**	**Sigmoid**	**IIA**	**well differentiated**
**CRC23**	**76**	**Male**	**Transverse**	**IIA**	**well differentiated**

Supplementary Figures S1–S5

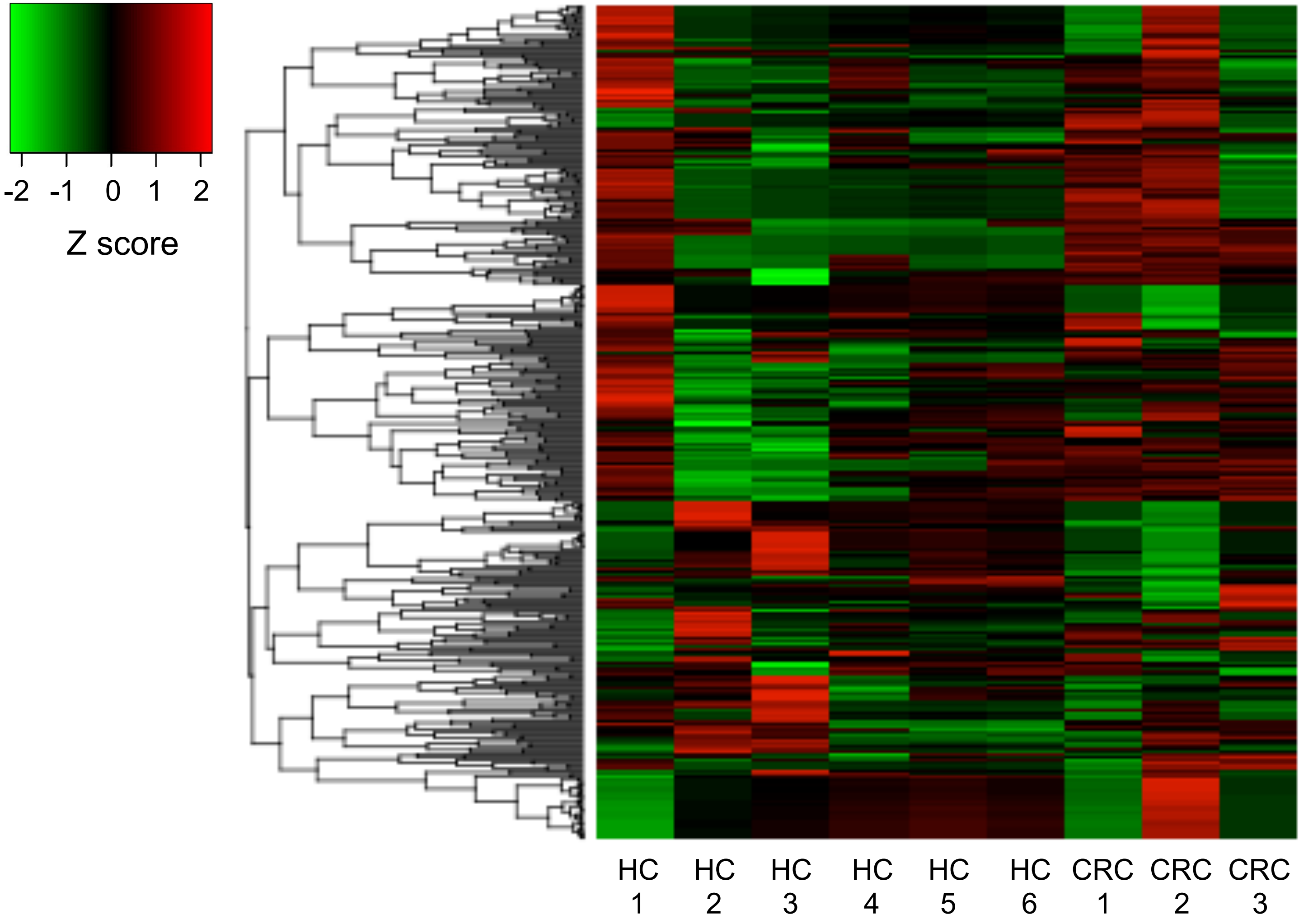

Supplementary Figure S1. Urinary miRNA microarray in the discovery cohort. HC, healthy control; CRC, colorectal cancer

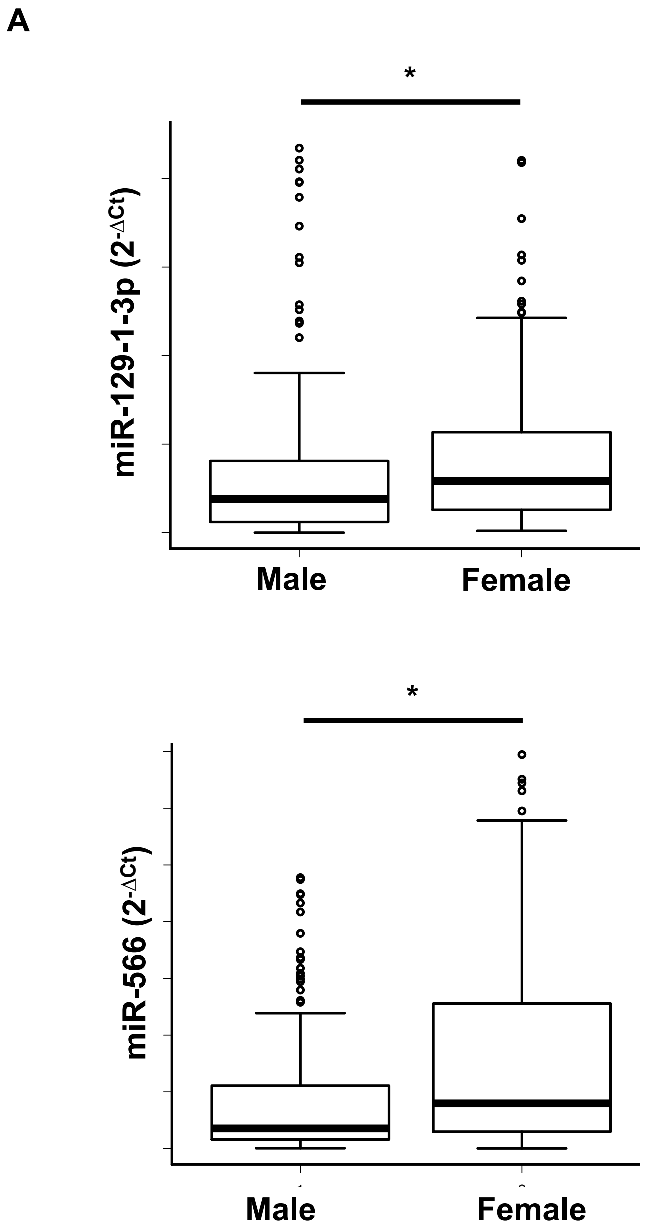

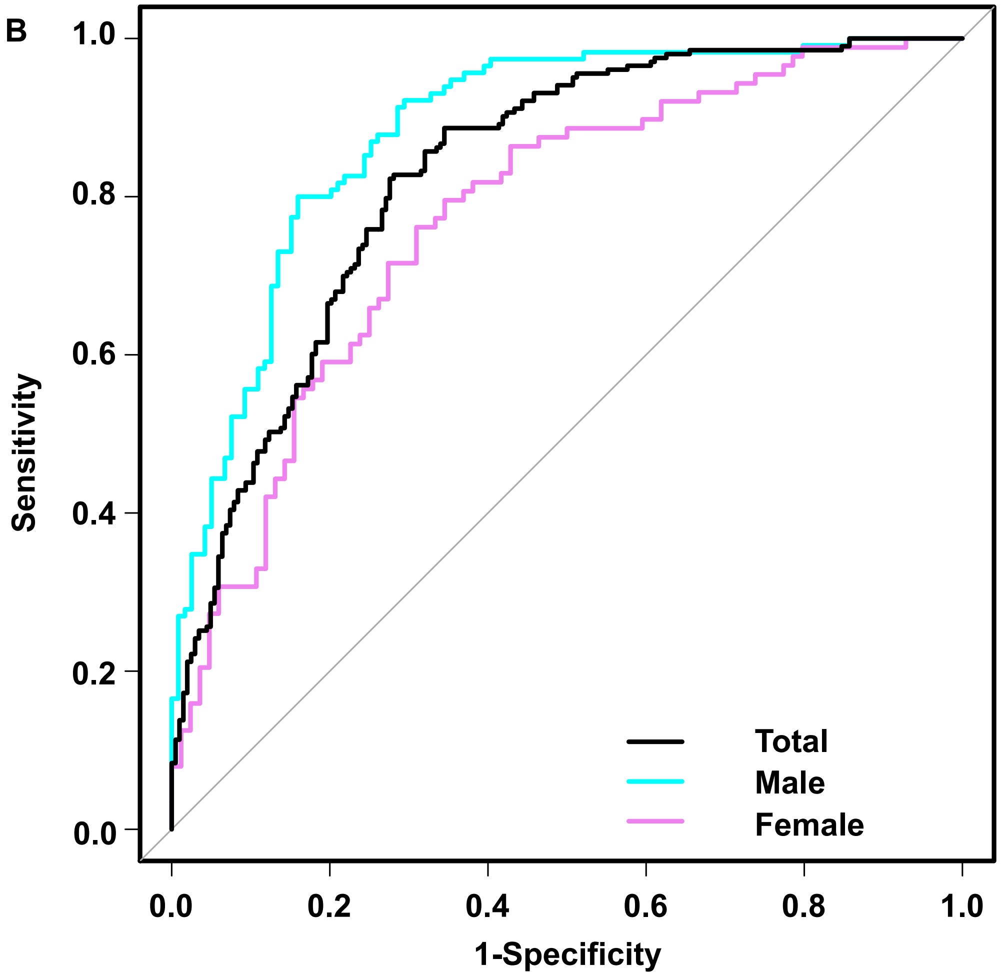

miR-129-1-3p + miR-566	AUC [95% CI]
Total	0.828 [0.789–0.868]
Male	0.882 [0.839–0.925]
Female	0.773 [0.703–0.843]

Supplementary Figure S2. Effect of gender on urinary biomarker. A. Boxplots according to genders, B. Receiver operator characteristics curve in the whole cohort.
AUC, area under the curve; 95% CI, 95% confidence interval; * p < 0.001

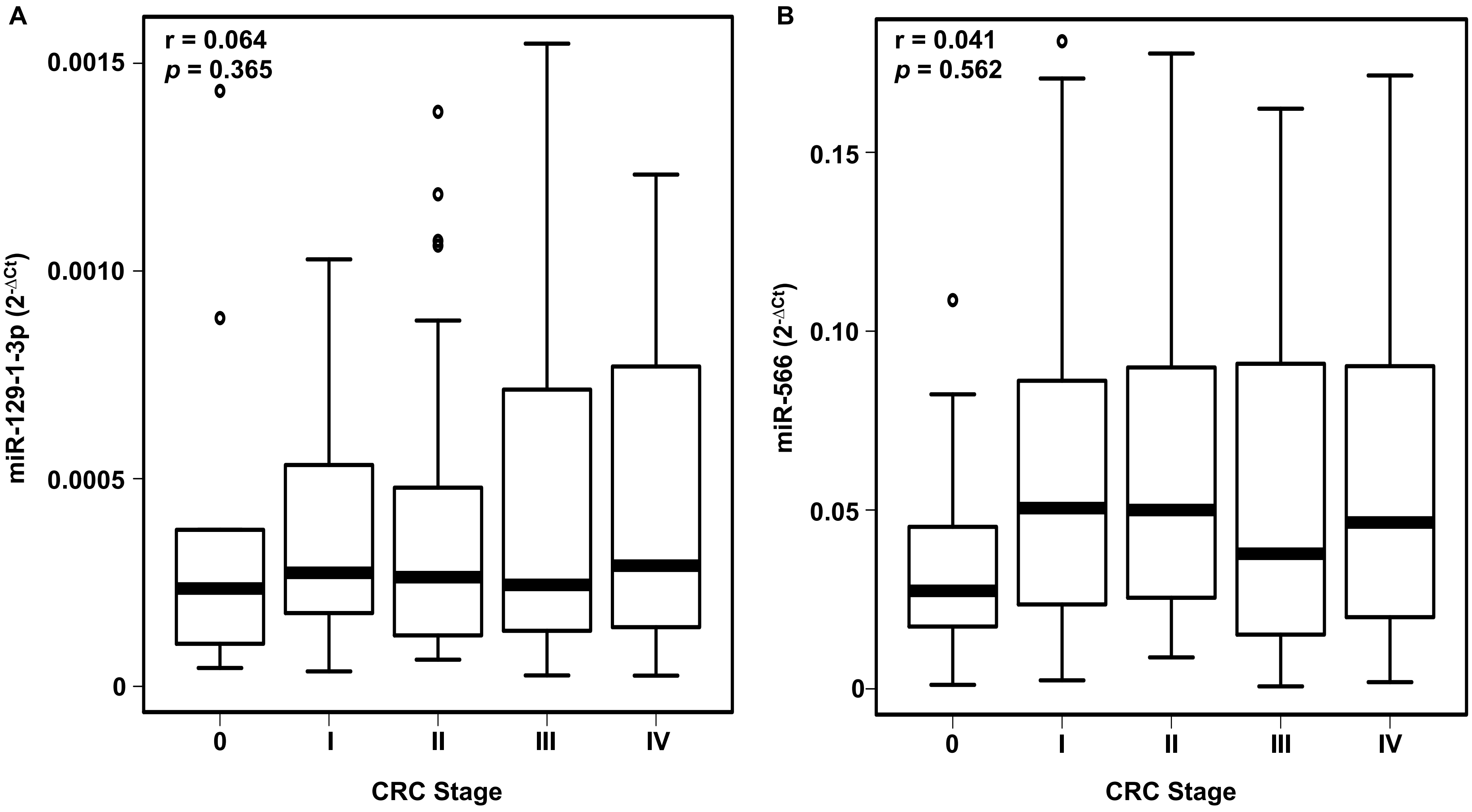

Supplementary Figure S3. Correlation between urinary miRNA level and disease stage. A. miR-129-1-3p. B. miR-566.

Ct, cycle threshold; CRC, colorectal cancer

Data were analyzed using the Spearman rank correlation.

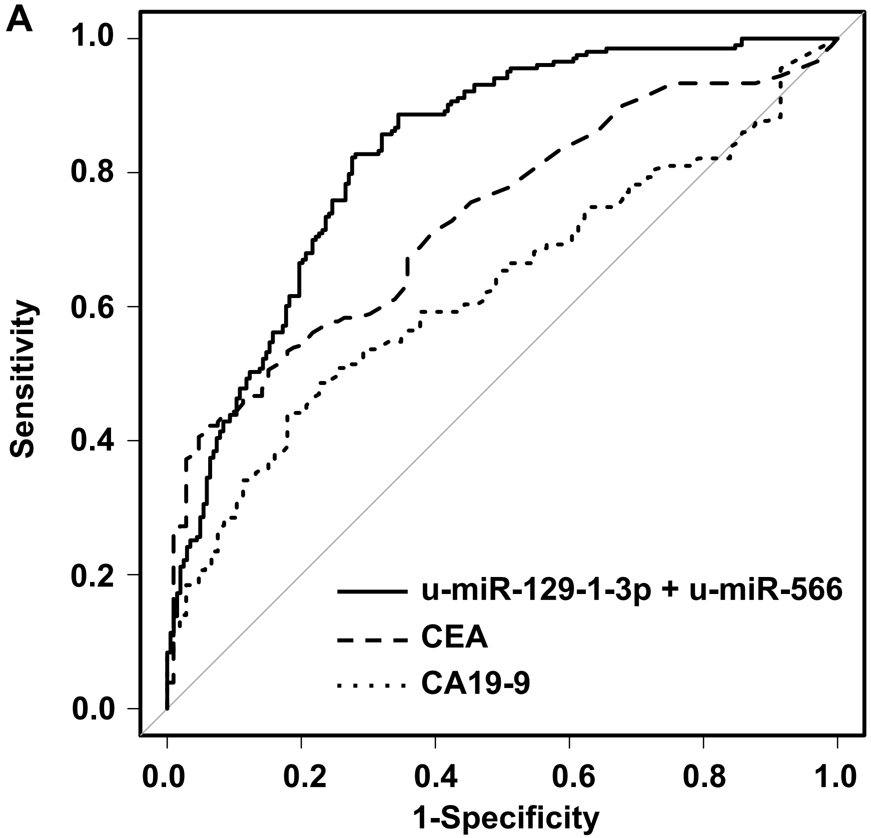

Biomarker	AUC [95% CI]	Sensitivity %	Specificity %
CEA	0.731 [0.674–0.789]	46.7*	87.7*
CA19-9	0.627 [0.562–0.691]	28.9*	90.6*
u-miR-129-1-3p + u-miR-566	0.828 [0.789–0.868]	82.8	71.9

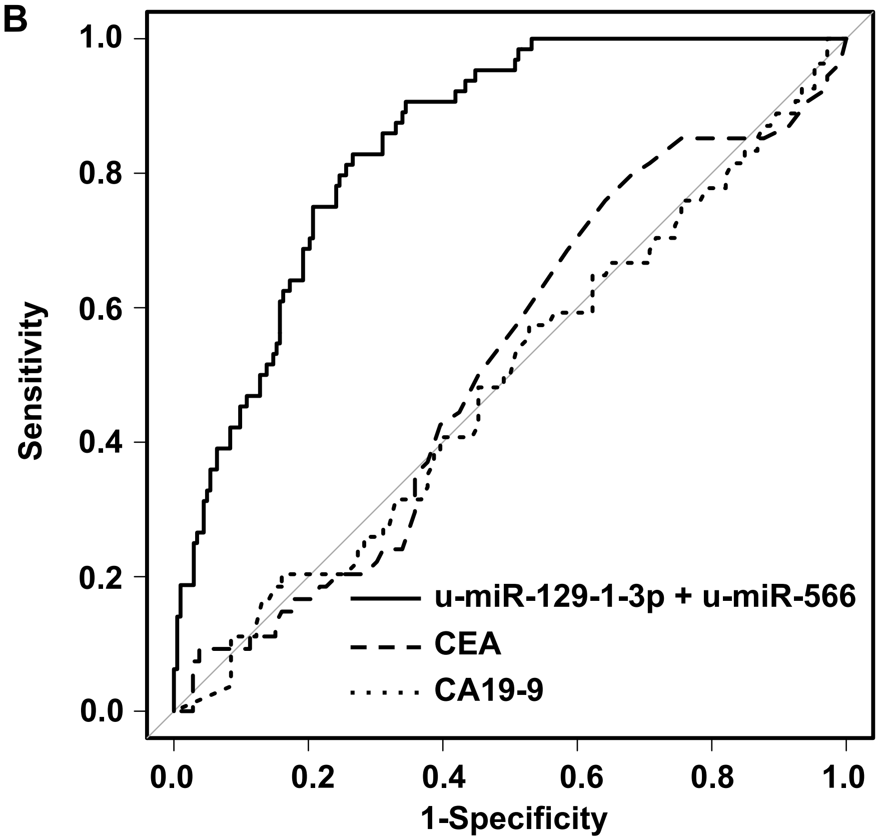

Biomarker	AUC [95% CI]	Sensitivity %	Specificity %
CEA	0.519 [0.425–0.613]	11.1*	87.7*
CA19-9	0.490 [0.395–0.586]	11.1*	90.6*
u-miR-129-1-3p + u-miR-566	0.845 [0.798–0.893]	82.8	73.4

Supplementary Figure S4 Comparison between urinary biomarker and serum CEA/CA19-9.
A. All Stage CRC. B. Stage 0/I CRC

* The cut-off values for CEA and CA19-9 were 5ng/mL and 37U/mL, respectively.

u-miR-129-1-3p, urinary miR-129-1-3p; u-miR-566, urinary miR-566; AUC, area under the curve; 95% CI, 95% confidence interval

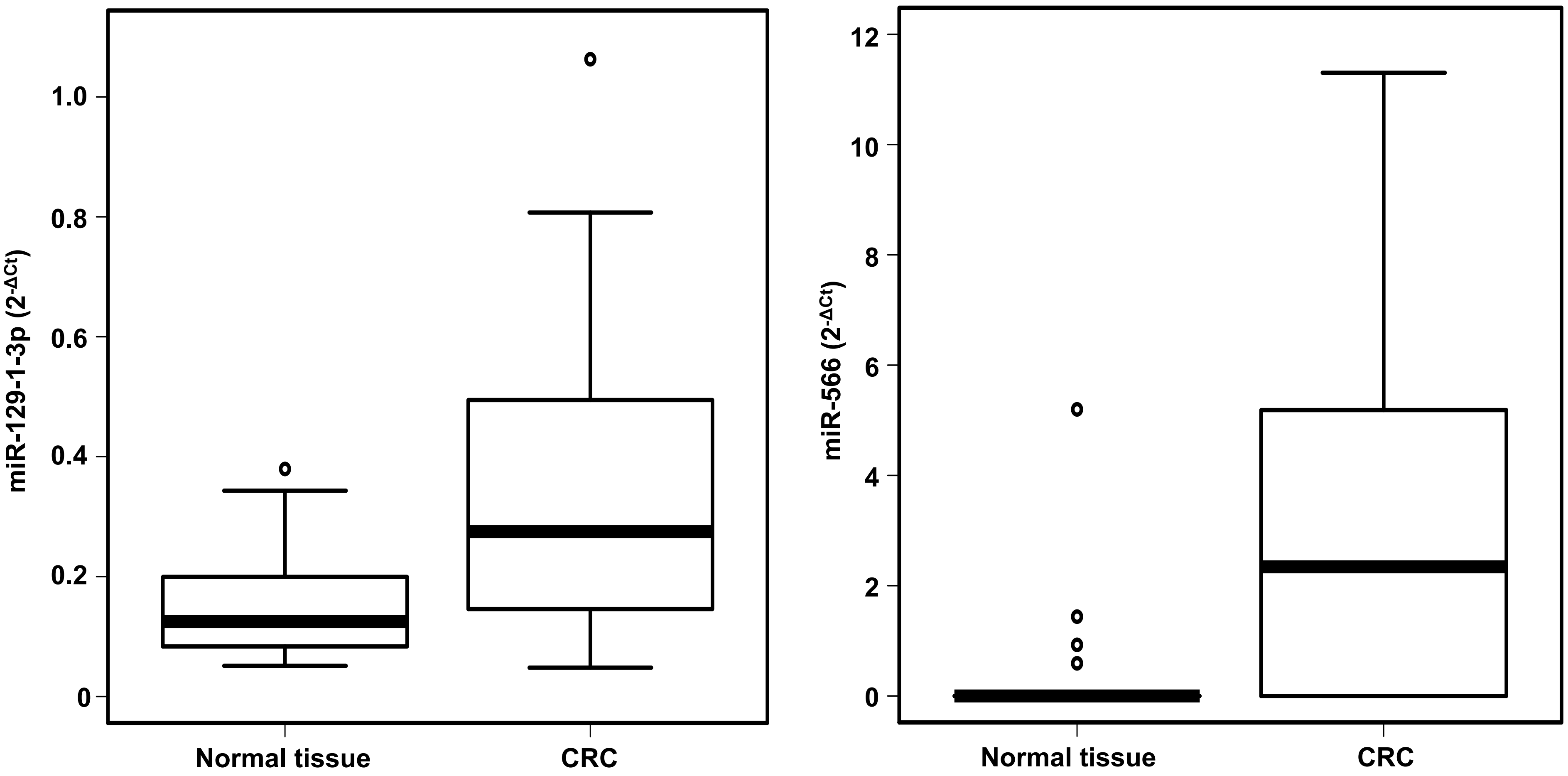

	$2^{-\Delta Ct}$ (Median, IQR)		*p* Value
	Normal tissue (*n* = 23)	CRC (*n* = 23)	
miR-129-1-3p	0.125<br>(0.083-0.199)	0.275<br>(0.146-0.494)	0.008
miR-566	0<br>(0-0)	2.342<br>(0-5.185)	0.002

Supplementary Figure S5. Expression of miR-129-1-3p and miR-566 in tissue sample.
Ct, cycle threshold; CRC, colorectal cancer; IQR, interquartile range
